# Epigallocatechin Gallate Stabilized by Cyclodextrin Inactivates Influenza Virus and Human Coronavirus 229E

**DOI:** 10.3390/microorganisms10091796

**Published:** 2022-09-06

**Authors:** Ryosuke Matsuura, Arisa Kawamura, Yasunobu Matsumoto, Yoshiki Iida, Masanori Kanayama, Masahiko Kurokawa, Yoko Aida

**Affiliations:** 1 Laboratory of Global Infectious Diseases Control Science, Graduate School of Agricultural and Life Sciences, The University of Tokyo, 1-1-1 Yayoi, Bunkyo-ku, Tokyo 113-8657, Japan; 2 Laboratory of Global Animal Resource Science, Graduate School of Agricultural and Life Sciences, The University of Tokyo, 1-1-1 Yayoi, Bunkyo-ku, Tokyo 113-8657, Japan; 3 HPG Co., Ltd., 3-18-9 Hatchobori, Chuo-ku, Tokyo 104-00332, Japan; 4 Graduate School of Clinical Pharmacy, Kyushu University of Health and Welfare, 1714-1 Yoshino-cho, Nobeoka, Miyazaki 882-8508, Japan

**Keywords:** epigallocatechin gallate, cyclodextrin, influenza virus, HCoV-229E, adsorption, inactivation

## Abstract

Natural products are attractive antiviral agents because they are environment-friendly and mostly harmless. Epigallocatechin gallate (EGCg), a type of catechin, is a well-known natural antiviral agent that can inhibit various viruses. However, EGCg easily oxidizes and loses its physiological activity. Although this problem can be overcome by combining EGCg with cyclodextrin (CD-EGCg), which makes it stable in water at high concentrations, the antiviral effect of this compound remains unclear. Here, we show that in Madin–Darby canine kidney (MDCK) and MRC-5 cells, CD-EGCg is cytotoxic for 50% of cells at 85.61 and 65.34 ppm, respectively. Furthermore, CD-EGCg mainly shows its antiviral effect during the adsorption step for all four influenza virus strains (median effect concentration (EC_50_) was 0.93 to 2.78 ppm). Its antiviral effect post-adsorption is less intense, and no inhibitory effect is observed on influenza viruses pre-adsorption. Moreover, human coronavirus 229E (HCoV-229E) was inhibited at the adsorption step in short contact (EC_50_ = 2.5 ppm) and long contact conditions (EC_50_ = 0.5 ppm) by mixing CD-EGCg with HCoV-229E. These results suggest that CD-EGCg effectively inhibits various viruses that require an adsorption step, and is an effective tool for preventing infection.

## 1. Introduction

In recent times, because of the spread of the global pandemic caused by severe acute respiratory syndrome coronavirus 2 (SARS-CoV-2), interest in public health measures to control the spread of viruses has increased. Various approaches for disinfection, including drugs and sterilization using light (e.g., ultraviolet [UV]) and photocatalysts, have been actively researched [1,2,3]. In addition, various vaccines and antiviral drugs have also been developed to prevent viral propagation and disease progression [4,5]. Natural products are attractive antivirals because they are environment-friendly and harmless.

Epigallocatechin gallate (EGCg), a type of catechin especially abundant in green tea, is a well-known natural antiviral product [6]. EGCg can inhibit many kinds of viruses, such as the influenza virus [7,8,9], human immunodeficiency virus (HIV) [10], hepatitis B virus (HBV) [11], human coronavirus (HCoV) [12], and SARS-CoV-2 [13]. In addition, EGCg inactivates the virus through various mechanisms. For example, it inhibits the adsorption and synthesis of the viral RNA of the influenza virus [7,8,9], inhibits DNA expression and replication of HBV [11], and inhibits protein interaction between SARS-CoV-2 and host cells [12]. EGCg interacts with viral proteins, DNA, and RNA, regulates cancer development, and is not toxic to humans [14]. It is easily oxidized and loses physiological activity upon oxidation [15]. Therefore, the instability of EGCg limits its effectiveness. However, inclusion in cyclodextrin enhances the stability of EGCg [16]. Because cyclodextrin is recognized as being completely safe, it is used in over 50 marketed pharmaceutical products for enhancing the solubility, improving the stability, and increasing the bioavailability of drugs [17,18]. In addition, the antioxidant activity of EGCg is enhanced rather than inhibited by its inclusion in cyclodextrin [19]. However, the antiviral effect of EGCg compounded with cyclodextrin has not been studied.

In this study, we investigated the antiviral effect of EGCg stabilized in water at high concentrations by cyclodextrin (CD-EGCg) against the influenza virus, a common and significant agent of human disease, and HCoV-229E, a virus closely related to SARS-CoV-2. In addition, we investigated the cytotoxicity of stabilized-EGCg against Madin–Darby canine kidney (MDCK) cells and MRC-5 cells, which are host to the influenza virus and HCoV-229E, respectively. To our knowledge, this is the first report showing that CD-EGCg exerts an antiviral effect on the influenza virus and HCoV-229E, and that its cytotoxicity to MDCK cells and MRC-5 cells is extremely low.

## 2. Materials and Methods

### 2.1. EGCg Stabilization

EGCg (Sunphenon; Taiyo Kagaku Co., Ltd., Tokyo, Japan) at a concentration of 10,000 ppm was stabilized in sterilized water containing 0.1% β-cyclodextrin, 0.4% γ-cyclodextrin, and 0.2% ascorbic acid. Sterilized water containing 0.1% β-cyclodextrin, 0.4% γ-cyclodextrin, and 0.2% ascorbic acid was used as negative control.

### 2.2. Cells

MDCK cells (RCB0995; Riken Bank, Wako, Japan) were cultured in Dulbecco’s modified Eagle’s medium (DMEM; Thermo Fisher Scientific, Waltham, MA, USA) containing penicillin, streptomycin, and glutamine (PSG; Gibco, Los Angeles, CA, USA) and 10% fetal bovine serum (FBS; Sigma-Aldrich, St. Louis, MO, USA). MRC-5 cells (CCL-171; ATCC, Manassas, VA, USA) were cultured in Eagle’s minimum essential medium (EMEM; Thermo Fisher Scientific) containing PSG and 10% FBS.

### 2.3. Stock Virus

Stocks of influenza virus A/Bangkok/93/03, influenza A/PR8/8/34, influenza A/Aichi/2/68, and influenza B/Singapore were propagated using MDCK cells [20]. The stock of HCoV-229E (VR-740; ATCC) was propagated using MRC-5 cells cultured in DMEM containing 2% FBS and 1% PSG, and titrated using 50% tissue culture infective dose (TCID_50_) assays.

### 2.4. Cell Viability Assay

Cell toxicity was measured as previously described using the WST-8 assay [21]. Briefly, MDCK cells or MRC-5 cells (1.0 × 10^5^ cells/well) were cultured in 24-well tissue culture plates for 17 h. CD-EGCg (diluted 0–100 ppm) was added to each well, and the plates were incubated at 37 °C for 48 h. After incubation, 30 μL of WST-8 reagent (Dojindo, Kumamoto, Japan) was added and incubated at 37 °C for 1.5 h. Finally, 100 μL of supernatant was transferred to a 96-well plate, and absorbance at 450 nm was measured using the microplate reader EnSight (PerkinElmer, Waltham, MA, USA). The untreated cells were defined as having 100% cell viability. Cytotoxicity for 50% of the cells (CC_50_) was calculated from linear regression of cell viability vs. CD-EGCg concentration.

### 2.5. Plaque Assay

The titer of influenza virus was measured using the plaque assay [20]. MDCK cells cultured in 60-mm dishes were infected with 200 μL of influenza virus at 4 °C for 1 h. After incubation, the cells were washed with phosphate-buffered saline (PBS), overlayed with 0.8% agarose gel (Ina Food Industry Co., Ltd., Nagano, Japan) to limit free virus spread, and incubated at 37 °C for 48 h. Following incubation, the cells were fixed with formalin, and the number of plaques was counted.

### 2.6. TCID_50_ Assay

The TCID_50_ assay was performed as previously described [2,3] to measure the titer of HCoV-229E. Briefly, MRC-5 cells were cultured in a 96-well plate (2 × 10^4^ cells per well), infected with 100 μL of 10-fold serially diluted HCoV-229E, and incubated at 37 °C for 5 days. Following incubation, viral infection in each well was determined based on the virus-induced cell cytopathic effect.

### 2.7. CD-EGCg Treatment for Influenza Virus

The antiviral effect of CD-EGCg on the influenza virus was confirmed using the plaque assay method [20] with a minor modification. For CD-EGCg treatment at pre-adsorption of influenza virus, the MDCK cells were treated either with 200 μL of 0, 1, 3, 10, or 30 ppm CD-EGCg or the solvent (sterilized water containing 0.1% β-cyclodextrin, 0.4% γ-cyclodextrin and 0.2% ascorbic acid) for 1 h. Then, 200 μL of 500 plaque forming units (PFU)/mL influenza virus were added after washing with PBS. After adsorption at 4 °C for 1 h, cells were overlayed on a 0.8% agarose gel and incubated for 2 days for plaque assay.

For CD-EGCg treatment during adsorption of the influenza virus, we added 200 μL of 500 PFU/mL influenza virus mixed with 0, 0.3, 1, 3, or 10 ppm of CD-EGCg to the MDCK cells. After adsorption at 4 °C for 1 h, the cells were washed by PBS, overlayed on a 0.8% agarose gel, and incubated for 2 days for plaque assay.

For CD-EGCg treatment after the adsorption of the influenza virus, the MDCK cells were added to 200 μL of 500 PFU/mL influenza virus. After adsorption at 4 °C for 1 h, the cells were washed with PBS, overlayed on a 0.8% agarose gel in the presence of 0, 1, 3, 10, or 30 ppm of CD-EGCg, and incubated for 2 days for plaque assay.

The median effect concentration (EC_50_) was calculated by regression analysis.

### 2.8. CD-EGCg Treatment for HCoV-229E

The antiviral effect of CD-EGCg on HCoV-229E was confirmed using the TCID_50_ method [2,3] with a minor modification. We mixed 225 μL of 250 TCID_50_/mL HCoV-229E with 25 μL of 0, 1, 2.5, 5, 10, 25, 50, or 100 ppm CD-EGCg and incubated the mixture for 30 s to mimic short contact conditions, and for 1 h to simulate long contact conditions. The mixture was serially diluted using PBS containing the same concentration of stabilized EGCg, and 100 μL of the mixture was added to the MRC-5 cells. The cells were incubated for 1 h at 4 °C and washed with PBS. The infectivity of HCoV-229E was measured using the TCID_50_ method. EC_50_ was calculated using regression analysis.

### 2.9. Statistical Analysis

Two-way analysis of variance (ANOVA) with Dunnett’s test was used to compare all the samples with a sample with 0 ppm CD-EGCg to determine statistical significance. All calculations were performed using R software (version 3.6.3, R Foundation for Statistical Computing, Vienna, Austria).

### 2.10. Reagents and Chemicals

The reagents and chemicals used in these experiments were the same as those described previously [2,3,20], unless otherwise stated.

## 3. Results

### 3.1. Cytotoxicity of Stabilized EGCg

**Figure 1:**
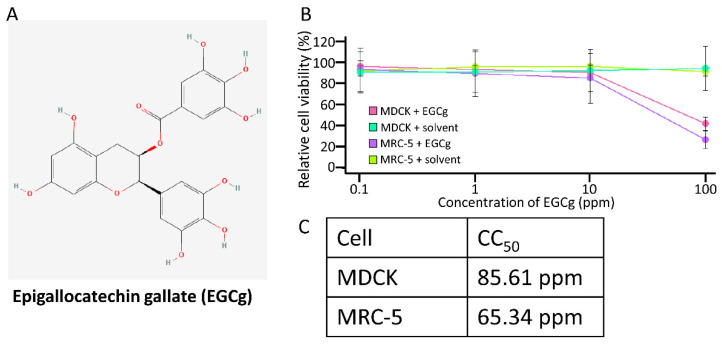
Cytotoxicity of CD-EGCg. (**A**) Chemical structure of stabilized [22]. (**B**) The cytotoxicity of CD-EGCg was measured by WST-8 assay. Assays were performed in two independent experiments. Relative cell viability was calculated according to the following equation: Relative cell viability = OD value at each concentration/OD value at 0 ppm. (**C**) Cytotoxicity for 50% of the cells (CC_50_) was calculated by regression analysis.

The cytotoxicity of CD-EGCg (Figure 1A) was examined in the MDCK cells and MRC-5 cells using the WST assay (Figure 1B). As shown in Figure 1C, cell viability was not reduced at any concentration up to 10 ppm CD-EGCg in both the MDCK and MRC-5 cells; a concentration of 100 ppm CD-EGCg remarkably decreased cell viability. By contrast, the solvent did not induce cytotoxicity in either the MDCK or MRC-5 cells. The CC_50_ of CD-EGCg was 85.61 ppm and 65.34 ppm in the MDCK and MRC-5 cells, respectively. These results suggested that CD-EGCg is cytotoxic at high concentrations.

### 3.2. Antiviral Activity of CD-EGCg against Influenza Virus

**Figure 2:**
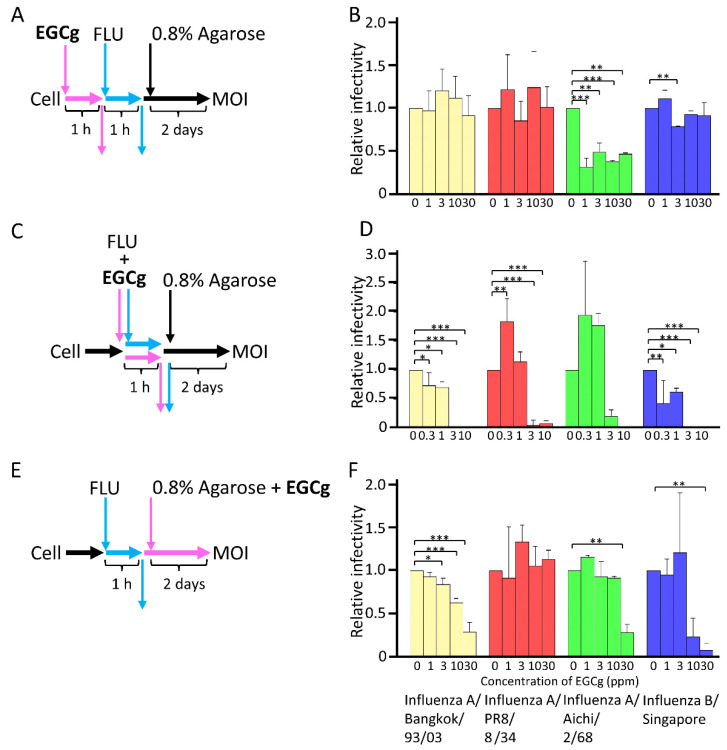
EGCg inactivation ability on influenza virus. (**A**) The inactivation ability of EGCg on influenza virus at pre-adsorption. Each concentration of CD-EGCg was added to MDCK cells and incubated for 1 h, and then removed. MDCK cells treated with CD-EGCg were adsorbed with 100 PFU for each influenza strain for 1 h and 0.8% agarose was overlaid. After 2 days incubation, the number of plaques was counted. (**B**,**D**,**F**) Each column and error bar represents the mean ± standard deviation (SD) of three independent experiments. All values in each group were compared with the 0 ppm sample by two-way ANOVA with Dunnett’s test. Asterisk indicates a statistically significant difference (* *p* < 0.05; ** *p* < 0.01; *** *p* < 0.001). (**C**) The inactivation ability of CD-EGCg on influenza virus. Each concentration of CD-EGCg was mixed with 100 PFU for each influenza strain. Then, the mixture was immediately added to MDCK cells and adsorbed for 1 h at 37 °C. After adsorption, cells were washed by PBS and 0.8% agarose was overlaid, and then incubated for 2 days, and the number of plaques was counted. (**E**) The inactivation ability of CD-EGCg on influenza virus post-infection. A total of 100 PFU for each influenza strain was adsorbed with MDCK cells for 1 h. After adsorption, cells were washed by PBS and 0.8% agarose containing each concentration of CD-EGCg was overlaid and incubated for 2 days, and the number of plaques was counted.

EGCg inhibits many kinds of viruses, such as the influenza virus [7,8,9], HIV [10], HBV [11], HCoV [12], and SARS-CoV-2 [13]. However, it is unclear whether CD-EGCg stabilized in water at high concentrations has an antiviral effect. Therefore, to clarify the antiviral ability and the point of action of CD-EGCg, three types of experiments were performed. We used CD-EGCg at a concentration of 30 ppm, which is lower than CC_50_ in MDCK and MRC-5 cells, as shown in Figure 1C. In the first experiment, CD-EGCg was added to MDCK cells 1 h post-infection (Figure 2A). After the pre-treatment, the cells were infected with the influenza virus, and the virus was adsorbed for 1 h. After adsorption, the cells were subjected to plaque assay, and the multiplicity of infection (MOI) of influenza virus was measured. As shown in Figure 2B, CD-EGCg treatment at the pre-adsorption stage had no inhibitory effect on influenza virus A/Bangkok/93/03, influenza A/PR8/8/34, and influenza B/Singapore. In the case of influenza virus A/Aichi/2/68, although CD-EGCg significantly decreased infectivity at 1 to 30 ppm, it did not yield inhibition levels up to about 70% or interfere in a dose-dependent manner. Thus, the EC_50_ was over 30 ppm for all influenza virus strains (Table 1), indicating that CD-EGCg did not exert an antiviral effect upon pre-treatment.

**Figure 3:**
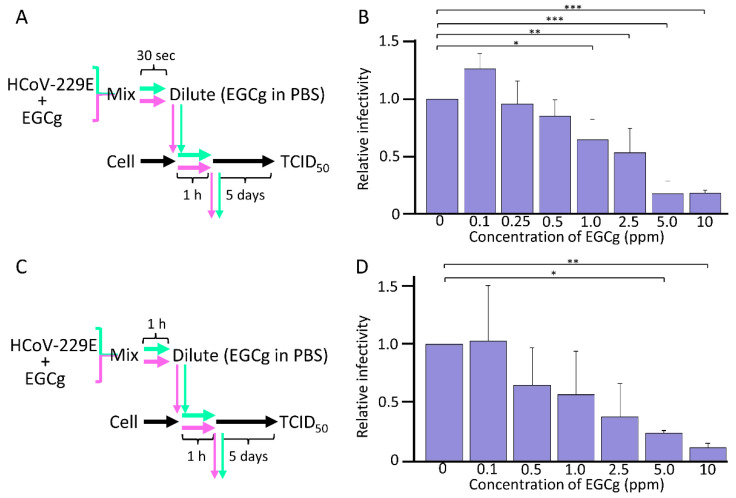
EGCg inactivation ability on HCoV-229E. (**A**,**C**) The inactivation ability of EGCg on HCoV-229E virus. Each concentration of EGCg was mixed with HCoV-229E (250 TCID_50_/_mL_) for 30 s or 1 h for short contact condition or long contact condition, respectively. The mixture was then 10-fold serial diluted by PBS containing same concentration of EGCg and added to MRC-5 cells. Cells were adsorbed for 1 h and washed by PBS. After 5 days of incubation, the infectivity of HCoV-229E was measured using the TCID_50_ method. (**B**,**D**) Each column and error bar represent the mean ± standard deviation (SD) of three independent experiments. All values in each group were compared with the 0 ppm sample by two-way ANOVA with Dunnett’s test. Asterisk indicates a statistically significant difference (* *p* < 0.05; ** *p* < 0.01; *** *p* < 0.001).

In the second experiment, CD-EGCg was added to MDCK cells during adsorption. CD-EGCg and the influenza virus were mixed and added to MDCK cells (Figure 2C). The MDCK cells were incubated for 1 h, washed with PBS, immersed in 0.8% agarose, and incubated for two days. As shown in Figure 2D, 3 and 10 ppm of CD-EGCg inhibited the replication of the influenza viruses regardless of the strains during the adsorption step. The EC_50_ of EGCg treatment was 1.41, 2.19, 2.78, and 0.93 ppm for influenza A/Bangkok/93/03, A/PR8/834, A/Aichi/2/68, and B/Singapore, respectively (Table 1).

In the third experiment, CD-EGCg was added to MDCK cells after adsorption. The MDCK cells were adsorbed with the influenza virus for 1 h, washed with PBS, immersed in 0.8% agarose containing CD-EGCg, and incubated for two days (Figure 2E). As shown in Figure 2F, CD-EGCg treatment after adsorption significantly interfered with the replication of influenza virus A/Bangkok/93/03 up to about 70% in a dose-dependent manner. Only 30 ppm of CD-EGCg significantly inhibited the replication of influenza A/Aichi/2/68 and influenza B/Singapore. However, CD-EGCg did not affect influenza A/PR8/8/34. Thus, the EC_50_ of CD-EGCg was 19.32, >30, 22.89, and 11.08 ppm for influenza A/Bangkok/93/03, A/PR8/834, A/Aichi/2/68, and B/Singapore, respectively (Table 1). Our results suggest that CD-EGCg in agarose gel inhibited the influenza virus that is released from the infected cells, but CD-EGCg exhibits reduced antiviral action during post-adsorption compared to during adsorption.

Thus, EGCg mainly shows its antiviral effect during and after the adsorption step. On the contrary, EGCg-treated MDCK cells did not resist infection from the influenza virus. Our results suggest that the antiviral effect of EGCg is exerted through direct contact with viruses.

### 3.3. Antiviral Activity of EGCg against HCoV-229E

The results stated above suggest that CD-EGCg mainly shows the antiviral effect during the adsorption step through direct contact between CD-EGCg and the virus. Therefore, to confirm whether CD-EGCg inhibits viruses other than influenza viruses, we exposed HCoV-229E directly to CD-EGCg. During the adsorption step, CD-EGCg was mixed with HCoV-229E for 30 s, and the mixture was immediately diluted with PBS containing EGCg. It was adsorbed to the MRC-5 cells, and the infectivity of HCoV-229E was determined using a TCID_50_ assay (Figure 3A). As shown in Figure 3B, the infectivity of HCoV-229E decreased upon contact with CD-EGCg in a dose-dependent manner. Next, to clarify the effect of the contact duration of CD-EGCg with the virus, the mixing time was extended up to 1 h (Figure 3C). As shown in Figure 3D, 5 and 10 ppm EGCg significantly inactivated HCoV-229E under a short contact duration. Interestingly, 0.5, 1.0, and 2.5 ppm CD-EGCg efficiently inactivated HCoV-229E under long contact duration compared to a short contact duration. The EC_50_ of EGCg for HCoV-229E was 2.5 and 0.5 ppm during short and long contact conditions, respectively (Table 1). These results suggest that the CD-EGCg exhibits an antiviral effect under long contact durations rather than short contact durations.

### 3.4. Point of Action of CD-EGCg

The EC_50_ of CD-EGCg treatment at the adsorption step was 1.41, 2.19, 2.78, and 0.93 ppm for influenza A/Bangkok/93/03, A/PR8/834, A/Aichi/2/68, and B/Singapore, respectively, indicating that this step showed the highest antiviral effect (Table 1). In addition, the EC_50_ of EGCg treatment post-adsorption was 19.32, >30, 22.89, and 11.08 ppm for influenza A/Bangkok/93/03, A/PR8/834, A/Aichi/2/68, and B/Singapore, respectively, suggesting that CD-EGCg acts during adsorption rather than post-adsorption. In contrast, if cells were treated with CD-EGCg before influenza virus infection, the EC_50_ was over 30 ppm regardless of the influenza virus strain, indicating that this step had no inhibitory effect on influenza viruses. These results are supported by findings for HCoV-229E; the EC_50_ of CD-EGCg for HCoV-229E was 2.5 and 0.5 ppm under short and long contact durations, respectively. These results suggest that CD-EGCg inactivates viruses through contact with the virus and inhibits the adsorption step.

## 4. Discussion

In this study, we demonstrated for the first time the antiviral effect of EGCg stabilized in water at high concentrations by cyclodextrin against the influenza virus and HCoV-229E. In particular, we showed that CD-EGCg inhibits virus adsorption upon direct contact, and inactivates the virus. This inhibition of virus adsorption by CD-EGCg was confirmed for two different viruses, the influenza virus and coronavirus, suggesting that CD-EGCg effectively inhibits various viruses that require an adsorption step. Mechanisms underlying the antiviral effect of EGCg was actively researched and EGCg was found to inhibit viruses by interacting with viral proteins, DNA, and RNA [7,8,9,10]. In particular, previous research shows that EGCg inhibits the adsorption of the influenza virus [7,8]. These studies support our findings showing the antiviral effect during the adsorption step through direct contact between CD-EGCg and the virus. Although we clarify that CD-EGCg inhibited adsorption, it remains unclear which proteins interact with CD-EGCg. Therefore, further experiments, such as pull-down assay, are needed to elucidate the detailed mechanism.

Our results showed that the cytotoxicity of CD-EGCg is very low. Indeed, the EC_50_ was sufficiently small compared to CC_50_, suggesting that CD-EGCg is an effective antiviral agent. EGCg is harmless and has high antiviral activity [6]. However, it is easily oxidized and loses its physiological activity on oxidation [15]. In this study, EGCg stabilized by cyclodextrin (CD-EGCg), which is a common additive for enhancing solubility, improving stability, and increasing bioavailability, had an antiviral effect similar to EGCg, and showed no sign of toxicity. Therefore, there are two main advantages of CD-EGCg. First, as it is highly concentrated and stable, CD-EGCg can be added to foods and drinks. Viral contamination can thus be prevented, and diseases can be prevented because of its physiological benefits, including its antioxidant and anti-inflammatory activity [23]. Second, since it inhibits viruses upon direct contact, viruses on contaminated surfaces can be inactivated by spraying CD-EGCg. Although ethanol and several other chemical compounds are useful for disinfecting contaminated surfaces, CD-EGCg is a suitable disinfectant because of its harmless nature.

In conclusion, to our knowledge, this is the first report showing that CD-EGCg has antiviral activity against the influenza virus and HCoV-229E, and demonstrating that CD-EGCg exerts an antiviral effect by direct contact during the adsorption step. Moreover, the study confirmed that the cytotoxicity of CD-EGCg is extremely low. Therefore, we propose that CD-EGCg is an effective tool for the prevention of infection.

## Figures and Tables

**Table 1:** EC_50_ of EGCg.

Virus	Strain	EGCg Treatment Pre-Adsorption	EGCg Treatment during Adsorption	EGCg Treatment Post-Adsorption
Influenza virus	A/Bangkok/93/03	>30	1.41 ± 0.17	19.32 ± 1.79
	A/PR8/8/34	>30	2.79 ± 0.09	>30
	A/Aichi/2/68	>30	2.78 ± 0.23	22.89 ± 1.44
	B/Singapore	>30	0.93 ± 0.35	11.08 ± 1.56
HCoV-229E		N. T. ^1^	2.5 (Short contact) 0.5 (Long contact)	N. T.

^1^ N. T. indicated not tested.

## Data Availability

Not applicable.
